# Addition of docetaxel to hormonal therapy in low- and high-burden metastatic hormone sensitive prostate cancer: long-term survival results from the STAMPEDE trial

**DOI:** 10.1093/annonc/mdz396

**Published:** 2019-09-27

**Authors:** N W Clarke, A Ali, F C Ingleby, A Hoyle, C L Amos, G Attard, C D Brawley, J Calvert, S Chowdhury, A Cook, W Cross, D P Dearnaley, H Douis, D Gilbert, S Gillessen, R J Jones, R E Langley, A MacNair, Z Malik, M D Mason, D Matheson, R Millman, C C Parker, A W S Ritchie, H Rush, J M Russell, J Brown, S Beesley, A Birtle, L Capaldi, J Gale, S Gibbs, A Lydon, A Nikapota, A Omlin, J M O'Sullivan, O Parikh, A Protheroe, S Rudman, N N Srihari, M Simms, J S Tanguay, S Tolan, J Wagstaff, J Wallace, J Wylie, A Zarkar, M R Sydes, M K B Parmar, N D James

**Affiliations:** 1 Department of Urology, The Christie and Salford Royal NHS Foundation Trusts, Manchester; 2 Genito-Urinary Cancer Research Group, Division of Cancer Sciences, The University of Manchester, Manchester; 3 MRC Clinical Trials Unit at UCL, Institute of Clinical Trials and Methodology, UCL, London; 4 London School of Hygiene and Tropical Medicine, London; 5 UCL Cancer Institute, London; 6 Guy’s and Saint Thomas’ NHS Foundation Trust, London; 7 St James University Hospital, Leeds; 8 Institute of Cancer Research, Sutton-London; 9 Department of Radiology, University Hospitals Birmingham NHS Foundation Trust, Birmingham; 10 Division of Cancer Sciences, The University of Manchester, Manchester; 11 Beatson West of Scotland Cancer Centre, University of Glasgow, Glasgow; 12 The Clatterbridge Cancer Centre NHS Foundation Trust, Liverpool; 13 Cardiff University, Cardiff; 14 Faculty of Education Health and Wellbeing, University of Wolverhampton, Wolverhampton; 15 Royal Marsden NHS Foundation Trust, London; 16 Institute of Cancer Sciences, Beatson West of Scotland Cancer Centre, Glasgow; 17 University of Sheffield, Sheffield; 18 Kent Oncology Centre, Maidstone; 19 Lancashire Teaching Hospitals NHS Foundation Trust, Preston; 20 Worcestershire Acute Hospitals NHS Trust, Worcester; 21 Portsmouth Oncology Centre, Queen Alexandra Hospital, Portsmouth; 22 Queen’s Hospital, Romford; 23 Torbay and South Devon NHS Foundation Trust, Torbay; 24 Sussex Cancer Centre, Brighton; 25 Department of Oncology and Haematology, Kantonsspital, St Gallen, Switzerland; 26 Centre for Cancer Research and Cell Biology, Queen’s University Belfast, Belfast; 27 East Lancashire Hospitals NHS Trust, Blackburn; 28 Oxford University Hospitals NHS Foundation Trust, Oxford; 29 Shrewsbury and Telford Hospital NHS Trust, Shrewsbury; 30 Hull and East Yorkshire Hospitals NHS Trust, Hull; 31 Velindre Cancer Centre, Cardiff; 32 Swansea University College of Medicine, Swansea; 33 The Christie NHS Foundation Trust, Manchester; 34 Heartlands Hospital, Birmingham; 35 Institute of Cancer and Genomic Sciences, University of Birmingham, Birmingham

**Keywords:** prostate cancer, metastatic, hormone naive, docetaxel, STAMPEDE trial, randomised control trial

## Abstract

**Background:**

STAMPEDE has previously reported that the use of upfront docetaxel improved overall survival (OS) for metastatic hormone naïve prostate cancer patients starting long-term androgen deprivation therapy. We report on long-term outcomes stratified by metastatic burden for M1 patients.

**Methods:**

We randomly allocated patients in 2 : 1 ratio to standard-of-care (SOC; control group) or SOC + docetaxel. Metastatic disease burden was categorised using retrospectively-collected baseline staging scans where available. Analysis used Cox regression models, adjusted for stratification factors, with emphasis on restricted mean survival time where hazards were non-proportional.

**Results:**

Between 05 October 2005 and 31 March 2013, 1086 M1 patients were randomised to receive SOC (*n* = 724) or SOC + docetaxel (*n* = 362). Metastatic burden was assessable for 830/1086 (76%) patients; 362 (44%) had low and 468 (56%) high metastatic burden. Median follow-up was 78.2 months. There were 494 deaths on SOC (41% more than the previous report). There was good evidence of benefit of docetaxel over SOC on OS (HR = 0.81, 95% CI 0.69–0.95, *P* = 0.009) with no evidence of heterogeneity of docetaxel effect between metastatic burden sub-groups (interaction *P* = 0.827). Analysis of other outcomes found evidence of benefit for docetaxel over SOC in failure-free survival (HR = 0.66, 95% CI 0.57–0.76, *P* < 0.001) and progression-free survival (HR = 0.69, 95% CI 0.59–0.81, *P* < 0.001) with no evidence of heterogeneity of docetaxel effect between metastatic burden sub-groups (interaction *P* > 0.5 in each case). There was no evidence that docetaxel resulted in late toxicity compared with SOC: after 1 year, G3-5 toxicity was reported for 28% SOC and 27% docetaxel (in patients still on follow-up at 1 year without prior progression).

**Conclusions:**

The clinically significant benefit in survival for upfront docetaxel persists at longer follow-up, with no evidence that benefit differed by metastatic burden. We advocate that upfront docetaxel is considered for metastatic hormone naïve prostate cancer patients regardless of metastatic burden.

**Key Message**

This report with long-term follow-up of M1 patients randomised within the STAMPEDE trial’s docetaxel comparison confirms a clinically significant benefit associated with the use of upfront docetaxel with androgen deprivation therapy in M1 prostate cancer. It should be a first-line option for fit metastatic hormone-naïve prostate cancer patients regardless of metastatic burden.

## Introduction

The primary analysis of STAMPEDE’s ‘docetaxel comparison’, reporting an improvement in survival, was triggered by reaching a pre-specified number of control group deaths [1]. The trial team agreed to update this analysis when there was a meaningful increase in the number of primary outcome measure events after further follow-up, expected to occur ∼3 years later. During that time, the Intermediate Clinical Endpoints in Cancer of the Prostate surrogacy work showed that measures based on metastatic progression could be used as a surrogate for survival in patients presenting with M0 disease, allowing trials in that setting to achieve increased power sooner [2]. Given the prognosis for metastatic and non-metastatic patients is now very different and that other trials of first-line docetaxel have kept metastatic and non-metastatic patients separate, the STAMPEDE team agreed that the long-term follow-up results would be analysed separately for these two groups of patients.

Since that initial STAMPEDE report, first-line systemic combination treatment options given with ADT in metastatic hormone naïve prostate cancer (mHNPC) have expanded to include abiraterone, enzalutamide and apalutamide as well as docetaxel [1, 3–8]. However there is still controversy about patient stratification and selection for treatment. Metastatic burden sub-group analyses of the CHAARTED and GETUG-15 trials have led some to conclude that docetaxel should not be given as a first-line treatment of patients presenting with ‘low metastatic burden’ disease [3, 9–11]. This represents ∼40% of patients presenting with *de novo* mHNPC [12, 13]. These were retrospective analyses of relatively small sub-groups of these trials and a number of groups have not been persuaded by these exploratory retrospective analyses. Reflecting this uncertainty, major treatment guidelines offer conflicting advice about whether all metastatic patients should receive combination treatment or whether this should be restricted only to those with ‘high-burden’, as specified in the CHAARTED trial [11, 14–16].

To address the hypothesis raised by CHAARTED, bone scans from the M1 docetaxel comparison cohort were collected retrospectively to determine the metastatic burden for STAMPEDE patients (independently of treatment assignment and outcome) and to undertake a stratified sub-group analysis. Outcome for the sub-groups categorised by individual metastatic burden was then determined using the extended patient follow-up now available in the STAMPEDE M1 cohort.

## Materials and methods

### Study design

The multi-arm, multi-stage STAMPEDE trial enrols patients with advanced high-risk or metastatic prostate cancer. Between 05 October 2005 and 31 March 2013, men with newly diagnosed metastatic prostate cancer were randomised on a 2 : 1 basis either to a standard-of-care (SOC) control group (‘Control’) or SOC + docetaxel treatment group (‘docetaxel’) [1]. SOC in M1 patients comprised long-term androgen deprivation therapy (ADT), the intervention being lifelong ADT and six cycles of docetaxel at standard dose. Randomisation followed a minimisation algorithm with a random element of 20%, stratified for hospital site, age at randomisation (<70 years old versus ≥70 years old at randomisation), WHO performance status (a score of 0 versus 1/2), nodal involvement (negative, positive or unspecified), planned ADT and usage (yes/no) of aspirin or other non-steroidal anti-inflammatory drugs (NSAIDs). The randomisation algorithm was developed and maintained centrally at the MRC Clinical Trials Unit at UCL. The trial was carried out in accordance with Good Clinical Practice guidelines, with full regulatory and ethical approval.

The first efficacy results for docetaxel within the STAMPEDE [1] used data from the initial randomisation in October 2005 to the data freeze with follow-up to March 2015. The date of this analysis was pre-determined by accumulation of events in the control group. In this paper, we report on a pre-planned long-term efficacy analyses of the M1 patient cohort but with updated results using extended follow-up data to July 2018.

### Procedures

All procedures relating to administration and reporting of docetaxel as a research treatment have been reported previously [1]. In brief, patients were randomised to lifelong ADT with or without six cycles of docetaxel (75 mg/m^2^) given 3-weekly with prednisolone 5 mg twice daily during the 18-week period of therapy.

The follow-up schedule, defined in the trial protocol, involved follow-up visits every 6 weeks until 6 months after randomisation, then 12 weeks to 2 years, 6 months to 5 years and annually thereafter. Toxicity was reported routinely at follow-up visits. Adverse event classification and grades followed the National Cancer Institute Common Terminology Criteria for Adverse Events (CTCAE) version 4.0. Since the primary report for this comparison, data on baseline metastatic burden has been retrospectively collected where possible for UK patients blinded to treatment outcomes. Metastatic burden was assessed using whole-body scintigraphy and computed tomography or magnetic resonance imaging staging scans, categorised following the definition used in the CHAARTED trial [3], where high-burden patients had either four or more bone metastases including one or more outside the vertebral body or pelvis, or any visceral metastases, or both. All other patients metastatic at baseline were categorised as having low metastatic burden according to this definition.

### Outcomes

Overall survival was specified as the primary efficacy outcome and defined as time from randomisation to death from any cause. Secondary outcomes for long-term efficacy data included: failure-free survival (FFS; time from randomisation to the first of any: biochemical, lymph node, distant metastatic progression or prostate cancer death); progression-free survival (PFS; time from randomisation to the first FFS event, not including biochemical progression); metastatic progression-free survival (mPFS; time from randomisation to either new metastases, progression of existing metastases or prostate cancer death) and prostate cancer-specific survival (PCSS; time from randomisation to prostate cancer death). Biochemical progression was assessed using prostate-specific antigen (PSA) measurements which were reported at each follow-up visit. The lowest PSA value within the first 24 weeks after enrolment was used to define a nadir, from which an increase of 50% and to a minimum level of 4 ng/ml indicated biochemical progression. For a small proportion of patients, PSA levels did not fall after enrolment and a nadir could not be estimated; these patients were considered to have biochemical progression at the date of randomisation. Patients without any event of interest reported by the time of the data freeze were censored in the analyses at the time they were last recorded in the trial as without an event.

The cause of death was assigned as prostate cancer/not prostate cancer where possible, using a set of rules pre-specified by the Trial Management Group’s End point Review Committee. Any deaths not meeting the pre-specified rules were individually reviewed by a trial clinician to assign a cause of death independent of the allocated treatment.

### Statistical analysis

Sample size calculations for trial design used the nstage function in Stata, based on a target HR of 0.75 for the primary efficacy analysis. Long-term outcome analysis was planned for ∼3 years after the first analysis, when there was projected to be a 40% increase in control group deaths. For the long-term analysis by metastatic disease burden, there was 66% and 77% power to detect a hazard ratio of 0.75 in the low and high-burden sub-groups, respectively. This calculation used the observed accrual and previous event rate in the control group (without reference to any accumulating differences between arms/sub-groups).

**Figure 1.**
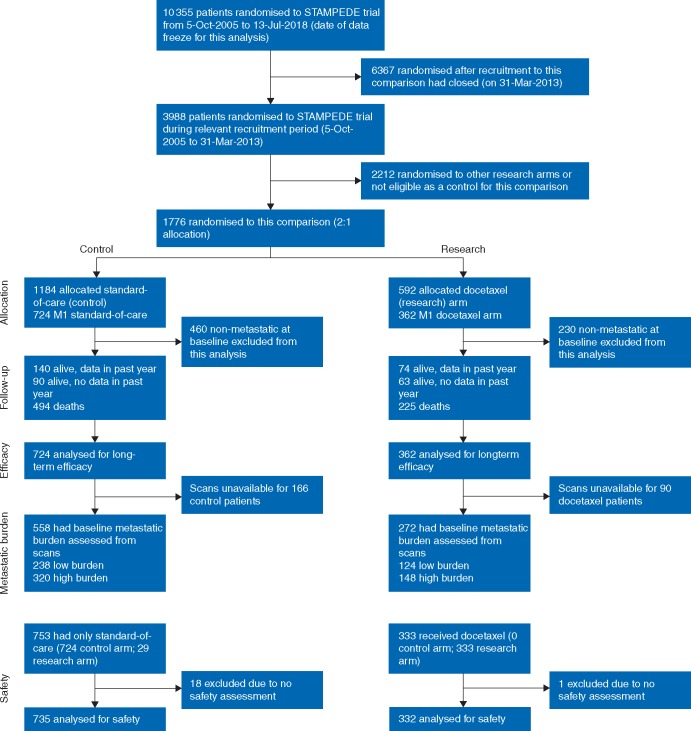
Consort diagram.

Efficacy analyses, following intention-to-treat principles, included all patients allocated to a trial arm. Toxicity and adverse event data are presented with patients grouped according to whether or not docetaxel treatment was reported as having been started (29 patients randomised to docetaxel who did not receive the drug are reported as Controls for the purposes of comparing toxicity between the treatments). See Figure 1 for full details of patients included in the analyses.

All time-to-event analyses followed standard survival analysis methods using Stata (version 15.1). Median duration of follow-up period was estimated using the Kaplan–Meier method with reverse-censoring of any reported deaths. Hazard ratios were estimated from Cox proportional hazards regression models, adjusted for minimisation factors used in the randomisation algorithm (nodal stage, age at randomisation, WHO performance score, use of aspirin/NSAIDs, planned use of SOC radiotherapy) and stratified by time period, as defined according to the other arms open to accrual in STAMPEDE (i.e. changes through closure or opening of other trial arms on the platform) or SOC practice changes. Non-parametric stratified log-rank tests were used to test for differences between the control and docetaxel groups, with stratification the same as that of the Cox regression models. Flexible parametric models were used to generate 5-year survival estimates, fitted using (5,5) degrees of freedom with adjustment variables as specified above. PCSS was analysed using Fine and Grey regression methods for competing risks analysis [17]. For all outcomes, models were tested for evidence of non-proportional hazards, and where found, interpretation of the results emphasises restricted mean survival time (RMST), which was calculated using a t* of 120 months, estimated as described previously [18]. For all statistical tests, two-sided tests were used and 95% confidence intervals and *P*-values are reported. Sub-group analyses are presented for all outcomes for metastatic burden sub-groups (low- and high-burden sub-groups). Although our emphasis is on metastatic burden, exploratory sub-group analyses are also presented for the primary outcome in order to give detail on the consistency of treatment effect across baseline factors of potential interest for this patient population: nodal status (N0, N+ or NX), Gleason sum score (≤7, 8–10 or unknown), patient age (<70 or ≥70) and WHO performance score (0 or 1–2).

## Results

One thousand and eighty-six metastatic patients (724 control/362 docetaxel) were recruited to STAMPEDE’s ‘docetaxel comparison’ between 05 October 2005 and 31 March 2013. The dataset for this analysis was frozen on 13 July 2018. As reported previously, baseline patient characteristics were well-balanced across trial arms (Table 1). We additionally report sub-group analyses according to metastatic burden, which was assessed using bone scans available from 830/1086 (76%) of all recruited patients. The patients included in these sub-group analyses were well-balanced across arms (Table 2 and supplementary Table S1, available at *Annals of Oncology* online). In addition, a comparison of Tables 1 and 2 demonstrates that the subset of patients included in the metastatic burden sub-group analyses was representative of the metastatic patients in the comparison as a whole, with the exception of the year of randomisation, where the patients included in the metastatic burden sub-group analyses were enrolled in the latter years of recruitment to the comparison. Figure 1 shows a Consort diagram with full details of patient numbers included in the analyses.

**Table 1.** Baseline characteristics for all metastatic patients, by trial arm

Patient characteristic	Control		Docetaxel	
Randomised (2 : 1 allocation)	724	100%	362	100%
Age at randomisation (years)				
Median	65		65	
IQR	60–71		60–70	
WHO performance status				
0	521	72%	270	75%
1–2	203	28%	92	25%
T stage				
T0	3	<1%	1	<1%
T1	12	2%	0	0%
T2	75	10%	51	14%
T3	404	56%	197	54%
T4	163	23%	82	23%
TX	67	9%	31	9%
Nodal status				
N0	242	33%	118	33%
N+	416	57%	211	58%
NX	66	9%	33	9%
Metastatic burden^a^				
Low	238	33%	124	34%
High	320	44%	148	41%
Unassessed	166	23%	90	25%
Site of metastases^b^				
Bone	634	88%	307	85%
Liver	15	2%	6	2%
Lung	33	5%	13	4%
Nodes^c^	221	31%	102	28%
Other	46	6%	25	7%
Gleason sum score				
≤7	158	22%	65	18%
8–10	480	66%	253	70%
Unknown	86	12%	44	12%
PSA				
Median	102.5		97	
IQR	32.8–354		40.5–340	
Time from diagnosis to randomisation (days)				
Median	69		73	
IQR	49–92		55–95	
Planned SOC RT^d^				
Not planned	677	94%	333	92%
Planned	47	6%	29	8%
Previously treated				
No	689	95%	347	96%
Yes	35	5%	15	4%
Pain from prostate cancer				
Absent	553	76%	270	75%
Present	154	21%	88	24%
Unknown	17	2%	4	1%
Year of randomisation				
2005	1	<1%	1	<1%
2006	28	4%	14	4%
2007	38	5%	19	5%
2008	70	10%	34	9%
2009	93	13%	45	12%
2010	111	15%	58	16%
2011	169	23%	87	24%
2012	172	24%	85	23%
2013	42	6%	19	5%
Total	724	100%	362	100%

a CHAARTED definition.

b Patients may have had more than one site of metastases at baseline, therefore are represented in more than one ‘site of metastases’ category. Percentages shown are per individual site for the total patients in the arm.

c Non-regional lymph nodes.

d Primary site RT was not standard-of-care (SOC) for M1 patients at the time of the trial. However, SOC RT was reported as planned for a small proportion of patients due to clinical decisions for these individual cases to receive RT to non-prostate locations, or due to mis-reporting of palliative RT.

**Table 2.** Baseline characteristics, for the subset of 830/1086 patients included in metastatic burden sub-group analysis, by trial arm

Patient characteristic	Control		Docetaxel	
Randomised	558	100%	272	100%
Age at randomisation (years)				
Median	65		65	
IQR	60–71		62–70	
WHO performance status				
0	405	73%	204	75%
1–2	153	27%	68	25%
T stage				
T0	1	<1%	1	<1%
T1	9	2%	0	0%
T2	56	10%	36	13%
T3	315	56%	155	57%
T4	129	23%	58	21%
TX	48	9%	22	8%
Nodal status				
N0	185	33%	88	32%
N+	322	58%	161	59%
NX	51	9%	23	8%
Metastatic burden^a^				
Low	238	43%	124	46%
High	320	57%	148	54%
Site of metastases^b^				
Bone	485	87%	226	83%
Liver	12	2%	4	1%
Lung	22	4%	12	4%
Nodes^c^	175	31%	78	29%
Other	38	7%	20	7%
Gleason sum score				
≤7	118	21%	51	19%
8–10	381	68%	188	69%
Unknown	59	11%	33	12%
PSA				
Median	102.5		96.8	
IQR	33–338.7		37.8–348.1	
Time from diagnosis to randomisation (days)				
Median	69		74	
IQR	50–92		56–95	
Planned SOC RT^d^				
Not planned	523	94%	252	93%
Planned	35	6%	20	7%
Previously treated				
No	528	95%	261	96%
Yes	30	5%	11	4%
Pain from prostate cancer				
Absent	427	77%	200	74%
Present	120	22%	69	25%
Unknown	11	2%	3	1%
Year of randomisation				
2005	0	0%	0	0%
2006	0	0%	0	0%
2007	0	0%	0	0%
2008	19	3%	8	3%
2009	82	15%	39	14%
2010	105	19%	52	19%
2011	157	28%	78	29%
2012	157	28%	78	29%
2013	38	7%	17	6%
Total	558	100%	272	100%

a CHAARTED definition.

b Patients may have had more than one site of metastases at baseline, therefore are represented in more than one ‘site of metastases’ category. Percentages shown are per individual site for the total patients in the arm.

c Non-regional lymph nodes.

d Primary site RT was not standard-of-care (SOC) for M1 patients at the time of the trial. However, SOC RT was reported as planned for a small proportion of patients due to clinical decisions for these individual cases to receive RT to non-prostate locations, or due to mis-reporting of palliative RT.

**Figure 2.**
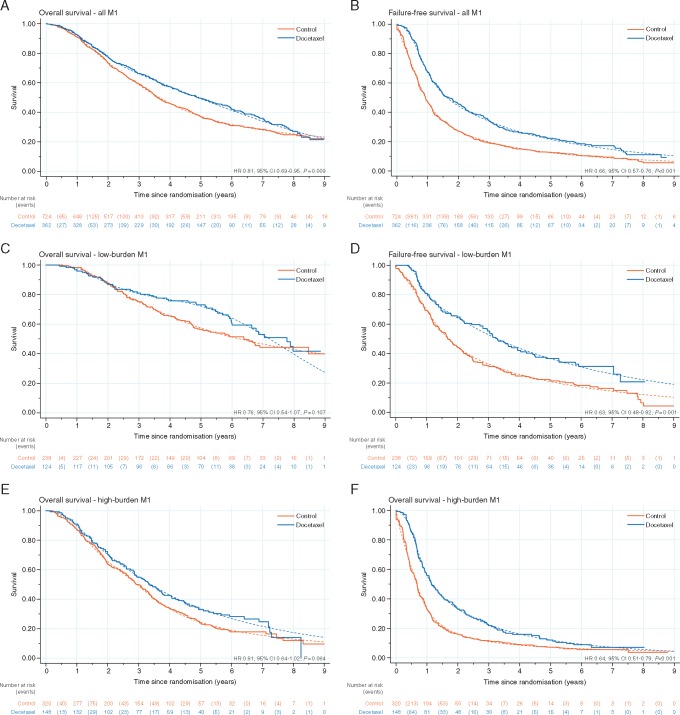
Kaplan–Meier curves (solid line) and fitted flexible parametric model estimates (dashed line) for overall survival (left) and failure-free survival (right), by trial arm, for all M1 patients (A,B) low-burden M1 patients (C,D) and high-burden M1 patients (E,F).

The median duration of follow-up was 78.2 months (inter-quartile range (IQR): 62.9–96.3). There were 719 deaths reported; 494/724 (68%) control patients died compared with 225/362 (62%) docetaxel patients. Control group patients had a median survival of 43.1 months and an estimated 5-year survival of 37% (95% CI 34% to 41%), whereas patients receiving docetaxel had a median survival of 59.1 months and 5-year survival of 49% (95% CI 44% to 54%). There was good evidence of a benefit from docetaxel on survival (stratified log-rank test *P* = 0.003, HR = 0.81, 95% CI 0.69–0.95; Figure 2A). As there was evidence (*P* = 0.016) of non-proportional hazards in the treatment effect, the interpretation of these results focuses on the difference in RMST between arms. This method showed evidence of a benefit of docetaxel, with an estimated difference of 6.0 months (95% CI 0.7–11.4) in RMST (over 120 months) between groups.

**Figure 3.**
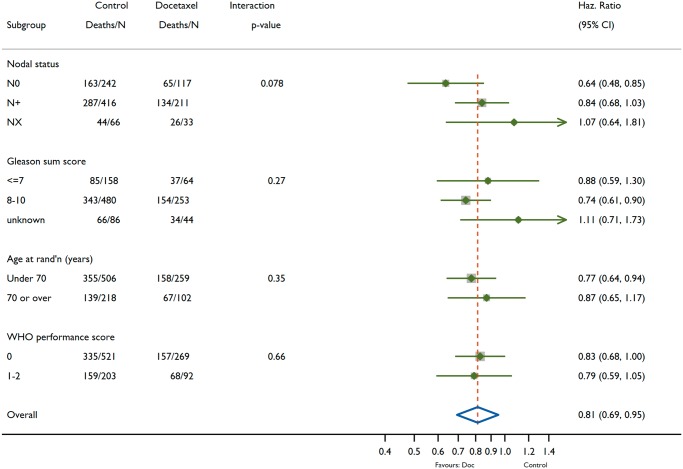
Effect of docetaxel on overall survival across exploratory sub-groups according to baseline factors.

There was no evidence of heterogeneity of treatment effect on survival over metastatic burden sub-groups (interaction *P* = 0.827). For the low-burden patients (*n* = 362, deaths = 166; Figure 2C), the median survival in control was 76.7 months, with 5-year survival 57% (95% CI 51% to 64%), compared with a median of 93.2 months and 5-year survival of 72% (95% CI 65% to 80%) for docetaxel. Results for high-burden patients (*n* = 468, deaths = 360) were similar in terms of docetaxel effect, although, as expected, the high-burden patients generally had shorter survival time than low-burden patients (Figure 2E). The median survival for control was 35.2 months and the 5-year survival estimate of 24% (95% CI 20% to 29%), compared with 39.9 months with a 5-year survival of 34% (95% CI 27% to 42%) for docetaxel. The hazard ratios were consistent in the low-burden (HR = 0.76, 95% CI 0.54–1.07, *P* = 0.107) and high-burden (HR = 0.81, 95% CI 0.64–1.02, *P* = 0.064) sub-groups. The consistency of docetaxel treatment effect on survival across other baseline characteristics was examined as exploratory sub-group analyses, summarised in Figure 3. There is no good evidence that the docetaxel effect varies across any of the sub-groups included (nodal status, Gleason sum score, age or WHO performance score).

**Figure 4.**
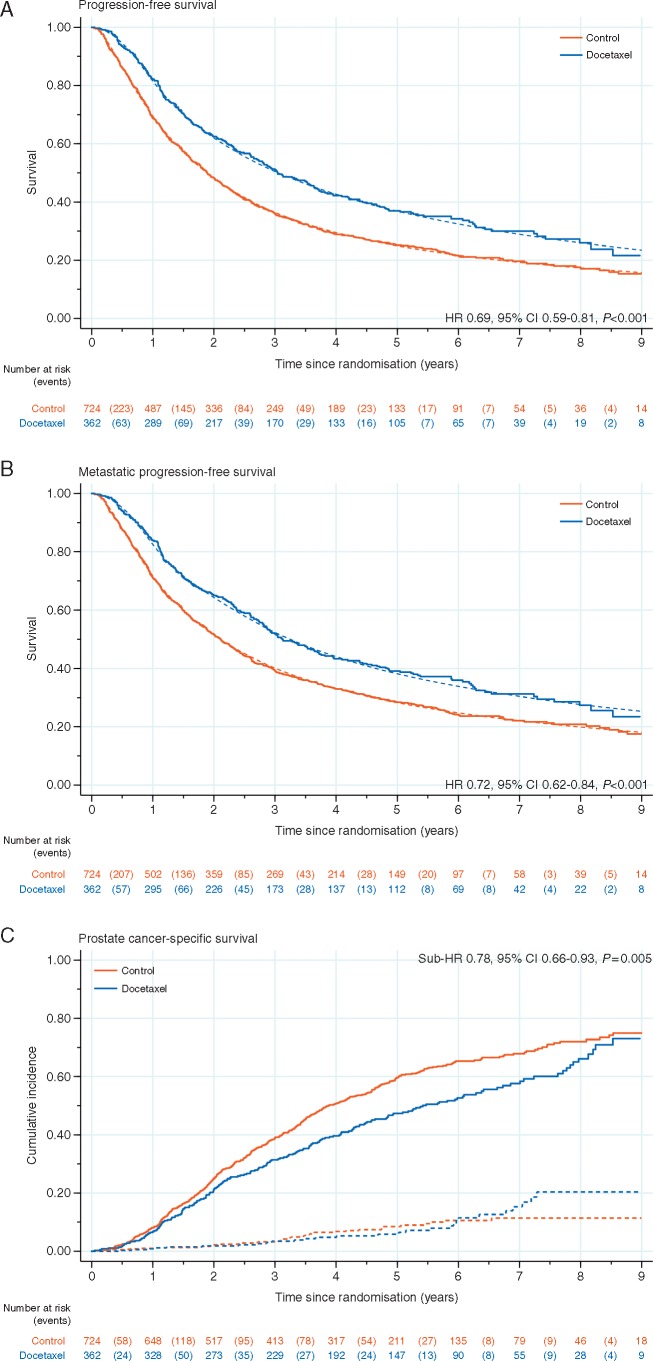
Kaplan–Meier curves (solid line) and fitted flexible parametric model estimates (dashed line), by trial arm, for (A) progression-free survival and (B) metastatic progression-free survival; (C) shows the cumulative incidence function, by trial arm, for prostate cancer death (solid line) and non-prostate cancer death (dashed line).

There was good evidence of significant benefit for adding docetaxel on failure-free (HR = 0.66, 95% CI 0.57–0.76, *P* < 0.001; Figure 2B), progression-free (HR = 0.69, 95% CI 0.59–0.81, *P* < 0.001; Figure 4A), metastatic progression-free (HR = 0.72, 95% CI 0.62–0.84, *P* < 0.001; Figure 4B) and PCSS (sub-HR = 0.78, 95% CI 0.66–0.93, *P* = 0.005; Figure 4C). Furthermore, the effect of treatment was consistent across metastatic burden sub-groups (interaction *P*-values: FFS = 0.792; PFS = 0.855; mPFS = 0.960; PCSS = 0.413) for other outcome measures (Table 3).

**Table 3.** Hazard ratio (95% CI) from adjusted Cox model, 5-year survival estimates by trial arm from flexible parametric model and restricted mean survival times (RMST) by trial arm, for each outcome measure and each metastatic burden sub-group

	Hazard ratio (95% CI)	5-year survival		RMST (months)			Interaction by metastatic burden *P*-value
		Control (%)	Docetaxel (%)	Control	Docetaxel	Difference (95% CI)	
Overall survival							
All patients	0.81 (0.69–0.95)	37	49	57.1	63.1	6.0 (0.7–11.4)	0.827
Low-burden	0.76 (0.54–1.07)	57	72	75.8	78.8	3.0 (−7.0 to 13.0)	
High-burden	0.81 (0.64–1.02)	24	34	44.8	51.3	6.6 (−0.8 to 14.0)	
Failure-free survival							
All patients	0.66 (0.57–0.76)	13	21	25.3	36.7	11.4 (6.8–16.0)	0.792
Low-burden	0.63 (0.48–0.82)	22	36	36.8	52.3	15.5 (6.2–24.8)	
High-burden	0.64 (0.51–0.79)	7	12	18.0	26.6	8.5 (2.7–14.4)	
Progression-free survival							
All patients	0.69 (0.59–0.81)	25	37	40.7	53.4	12.8 (7.2–18.3)	0.855
Low-burden	0.62 (0.45–0.85)	41	56	58.7	76.2	17.5 (7.4–27.6)	
High-burden	0.68 (0.54–0.85)	13	24	27.9	40.5	12.6 (5.3–19.8)	
Metastatic progression-free survival							
All patients	0.72 (0.62–0.84)	28	38	44.1	55.3	11.2 (5.8–16.6)	0.960
Low-burden	0.67 (0.48–0.92)	48	61	64.6	78.0	13.4 (2.9–23.9)	
High-burden	0.70 (0.56–0.87)	15	24	29.7	41.1	11.4 (3.9–18.9)	
Prostate cancer-specific survival^a^							
All patients	0.78 (0.66–0.93)	41	53	60.3	67.9	7.6 (1.9–13.4)	0.413
Low-burden	0.67 (0.45–0.98)	63	76	80.3	88.5	8.2 (−1.5 to 18.0)	
High-burden	0.84 (0.66–1.07)	27	36	47.1	54.6	7.5 (−0.4 to 15.3)	

a Competing risks model used to estimate hazard ratios for prostate cancer-specific survival, as described in the Materials and methods section.

Treatment adherence to docetaxel was reported previously [1]; all patients had completed docetaxel treatment before the first efficacy analysis. Twenty-nine metastatic patients allocated to Docetaxel never reported starting chemotherapy. They are included in Control for toxicity analysis (see Figure 1) which is summarised in Table 4 and supplementary Table S2 and Figure S1, available at *Annals of Oncology* online. A comparison of toxicity reported across groups in the first year of follow-up shows higher toxicity in docetaxel (42% docetaxel reported G3-5 toxicity versus 24% in Control). However, toxicity reports for subsequent follow-up, after the initial year, are balanced across groups (27% docetaxel reported G3-5 toxicity compared with 28% control), with no good evidence of increased toxicity in the docetaxel group after the first year of follow-up. Table 5 shows some evidence that control patients were more likely to report starting second-line (or subsequent) treatment following disease progression, with 80% reporting starting at least one further line of treatment compared with 68% for Docetaxel. The types of further therapy reported are broadly similar between the two groups.

**Table 4.** Worst adverse event grade reported per patient (across all CTCAE categories) for (i) up to one year on the trial; and (ii) after one year on the trial

Worst AE grade	Up to one year^a^				After one year^a^			
	Control		Docetaxel		Control		Docetaxel	
0	21	3%	0	0%	26	6%	12	5%
1	267	36%	63	19%	147	32%	65	25%
2	268	36%	128	39%	155	34%	109	43%
3	157	21%	96	29%	110	24%	63	25%
4	20	3%	44	13%	15	3%	6	2%
5	2	<1%	1	<1%	1	<1%	0	0%
No FU/SAE reported	18	n/a	1	n/a	18	n/a	1	n/a
Not on FU after one year	n/a	n/a	n/a	n/a	281	n/a	77	n/a
Total^b^	753	100%	333	100%	753	100%	333	100%

a Timed from randomisation.

b Total numbers shown for safety population, where 29 patients allocated to the Docetaxel Group never started Docetaxel treatment and are therefore included in the standard-of-care (SOC) group for safety reporting. Note that ‘missing’ data refers to patients who did not report AE data after this point (either died or withdrawn from the trial, or not reporting AEs after disease progression as specified in the trial protocol).

**Table 5.** Numbers of patients reporting starting second-line treatment (SLT), by trial arm

	Control		Docetaxel	
Randomised	724	100%	362	100%
Progression reported^a^	641	89%	291	80%
Any SLT reported	578	80%	246	68%
Life-prolonging treatments^b^				
Docetaxel	299	52%	49	20%
Abiraterone	196	34%	91	37%
Enzalutamide	113	20%	51	20%
Cabazitaxel	36	6%	29	11%
Radium-223	36	6%	21	9%
Other chemotherapy^c^	14	2%	12	5%
Other treatments^b^				
Anti-androgens	451	78%	178	72%
Dexamethasone	132	23%	49	20%
Zoledronic acid	130	22%	41	17%
Prednisolone/prednisone	97	17%	28	11%
Stilboestrol	85	15%	41	17%
Other bisphosphonate^d^	16	3%	7	3%
Strontium	12	2%	2	<1%

a Any progression event, including biochemical progression.

b Percentages shown are calculated using the total numbers of patients reporting at least one SLT for the relevant trial arm. Patient numbers only include patients confirmed to have progressed. Note that patients reporting more than one type of second-line treatment will be represented in more than once.

c Other chemotherapy, excluding Docetaxel and Cabazitaxel, which are shown separately.

d Other bisphosphonate, excluding Zoledronic acid, which is shown separately.

A sensitivity analysis was undertaken without the M1 patients retrospectively found not to have met all of the strict protocol eligibility criteria, mostly concerning a review of baseline blood pressure measurements. Removing these 120 patients (11%) did not change the primary outcome measure results HR = 0.81 (95% CI 0.68–0.96; *P* = 0.013).

## Discussion

In this updated long-term analysis, the relative survival benefit for adding docetaxel to ADT in mHNPC confirmed our previous findings. A statistically and clinically significant improvement in survival and a delayed time to metastatic progression was demonstrated with the combination treatment compared with ADT alone. Importantly, this benefit is seen irrespective of metastatic burden, with no evidence of heterogeneity between the low and high metastatic burden sub-groups across any outcome measures. This reinforces the principle that ADT and docetaxel can be considered as an effective first-line treatment option for men with mHNPC regardless of metastatic burden.

Two other trials have also evaluated the combination of docetaxel with ADT over ADT alone in mHNPC. The first trial, GETUG-AFU-15, enrolled 385 patients and reported with median follow-up of 84 months. This showed no clear evidence of improvement in survival by adding docetaxel to ADT over ADT alone (HR = 0.88, 95% CI 0.68–1.14, *P* = 0.3) [19]. The second trial, CHAARTED, enrolled 790 patients and reported with median follow-up of 54 months, demonstrating clear evidence of an improvement in survival for adding docetaxel (HR = 0.72, 95% CI 0.59–0.89, *P* = 0.0018) [3, 9]. STAMPEDE is the largest of the 3 trials, enrolling 1086 mHNPC patients and reporting here with median follow-up of 78 months. Our long-term results for these metastatic patients lie between those of GETUG-AFU-15 and CHAARTED and show clear evidence of improved survival associated with the combination of docetaxel + ADT over ADT alone (HR = 0.81, 95% CI 0.69–0.95, *P* = 0.009).

However, in contrast to the CHAARTED and GETUG-15 trials, we found no good evidence in this study of a difference in benefit between the high and low metastatic burden sub-groups for survival and all other outcome measures. Indeed, the point estimate of the benefit for ‘low-burden’ patients was of ‘greater’ magnitude than that for the high-burden group. Prior sub-group analysis conducted as part of the CHAARTED and GETUG-15 trials suggested a smaller overall survival benefit associated with the combination of docetaxel + ADT in patients with low metastatic burden compared with high metastatic burden [10]. Based on this, docetaxel was recommended by those authors as a first-line option only for high but not low-burden mHNPC [11]. That view was not universally accepted and the inherent limitations of the previous sub-group analyses can possibly explain the discordance to our new findings [14–16]. Nearly 25% of the patients enrolled in the other two trials presented with metastatic disease after previous radical treatment. That group of patients has a different natural history to those presenting with *de-novo* M1 disease [20, 21]. Consequently, the low metastatic burden sub-groups in the CHAARTED and the GETUG-15 trials had fewer than 160 *de-novo* mHNPC patients each [10]. In our updated report, ∼95% of patients had *de-novo* M1, with a total of 362 patients in the low metastatic burden sub-group. This larger sample size provides a stronger basis for estimating a treatment effect in this sub-group. Furthermore, there was no evidence of a difference in benefit associated with docetaxel with ADT over ADT alone when considering metastatic burden (interaction *P* = 0.827). Based on these findings, the combination of docetaxel with ADT should be a first-line treatment option for newly diagnosed mHNPC patients regardless of metastatic burden.

Our results have to be interpreted in light of more recently-recruited trials. Since the last report, a number of other systemic treatments such as abiraterone, apalutamide and enzalutamide have also shown statistically and clinically significant survival benefits over SOC alone when used as first-line agents in mHNPC [4–7]. None of the trials using androgen receptor pathway targeting have shown any evidence of heterogeneity of effect by metastatic burden. Our previous analysis of patients randomised contemporaneously within STAMPEDE to a docetaxel group or an abiraterone group did not show any difference in overall survival. Whilst that was acknowledged as under-powered, opportunistic comparison, it remains the only direct randomised comparison of docetaxel and abiraterone, and the results are in keeping with both agents being valid first-line options when combined with ADT [22].

## Conclusion

This updated report, with long-term follow-up and metastatic burden sub-group analysis, reinforces the benefits of adding docetaxel to ADT in mHNPC. The combination treatment prolongs survival and improves a range of outcome measures, including time to metastatic progression and subsequent therapy. It should be considered as a first-line option for fit patients with mHNPC, irrespective of metastatic burden.

## Supplementary Material

mdz396_Supplementary_Data
