# Corrigendum to Addition of docetaxel to hormonal therapy in low- and high-burden metastatic hormone sensitive prostate cancer: long-term survival results from the STAMPEDE trial: Ann Oncol 2019; 30: 1992–2003

**DOI:** 10.1016/j.annonc.2020.01.002

**Published:** 2020-01-31

**Authors:** N.W. Clarke, A. Ali, F.C. Ingleby, A. Hoyle, C.L. Amos, G. Attard, C.D. Brawley, J. Calvert, S. Chowdhury, A. Cook, W. Cross, D.P. Dearnaley, H. Douis, D. Gilbert, S. Gillessen, R.J. Jones, R.E. Langley, A. MacNair, Z. Malik, M.D. Mason, D. Matheson, R. Millman, C.C. Parker, A.W.S. Ritchie, H. Rush, J.M. Russell, J. Brown, S. Beesley, A. Birtle, L. Capaldi, J. Gale, S. Gibbs, A. Lydon, A. Nikapota, A. Omlin, J.M. O’Sullivan, O. Parikh, A. Protheroe, S. Rudman, N.N. Srihari, M. Simms, J.S. Tanguay, S. Tolan, J. Wagstaff, J. Wallace, J. Wylie, A. Zarkar, M.R. Sydes, M.K.B. Parmar, N.D. James, the STAMPEDE investigators

**Affiliations:** 1 Department of Urology, The Christie and Salford Royal NHS Foundation Trusts, Manchester; 2 Genito-Urinary Cancer Research Group, Division of Cancer Sciences, The University of Manchester, Manchester; 3 MRC Clinical Trials Unit at UCL, Institute of Clinical Trials and Methodology, UCL, London; 4 London School of Hygiene and Tropical Medicine, London; 5 UCL Cancer Institute, London; 6 Guy’s and Saint Thomas’ NHS Foundation Trust, London; 7 St James University Hospital, Leeds; 8 Institute of Cancer Research, Sutton-London; 9 Department of Radiology, University Hospitals Birmingham NHS Foundation Trust, Birmingham; 10 Division of Cancer Sciences, The University of Manchester, Manchester; 11 Beatson West of Scotland Cancer Centre, University of Glasgow, Glasgow; 12 The Clatterbridge Cancer Centre NHS Foundation Trust, Liverpool; 13 Cardiff University, Cardiff; 14 Faculty of Education Health and Wellbeing, University of Wolverhampton, Wolverhampton; 15 RoyalMarsden NHS Foundation Trust, London; 16 Institute of Cancer Sciences, Beatson West of Scotland Cancer Centre, Glasgow; 17 University of Sheffield, Sheffield; 18 Kent Oncology Centre, Maidstone; 19 Lancashire Teaching Hospitals NHS Foundation Trust, Preston; 20 Worcestershire Acute Hospitals NHS Trust, Worcester; 21 Portsmouth Oncology Centre, Queen Alexandra Hospital, Portsmouth; 22 Queen’s Hospital, Romford; 23 Torbay and South Devon NHS Foundation Trust, Torbay; 24 Sussex Cancer Centre, Brighton; 25 Department of Oncology and Haematology, Kantonsspital, St Gallen, Switzerland; 26 Centre for Cancer Research and Cell Biology, Queen’s University Belfast, Belfast, UK; 27 East Lancashire Hospitals NHS Trust, Blackburn, UK; 28 Oxford University Hospitals NHS Foundation Trust, Oxford, UK; 29 Shrewsbury and Telford Hospital NHS Trust, Shrewsbury, UK; 30 Hull and East Yorkshire Hospitals NHS Trust, Hull, UK; 31 Velindre Cancer Centre, Cardiff, UK; 32 Swansea University College of Medicine, Swansea, UK; 33 The Christie NHS Foundation Trust, Manchester, UK; 34 Heartlands Hospital, Birmingham, UK; 35 Institute of Cancer and Genomic Sciences, University of Birmingham, Birmingham, UK

The authors regret that Fig.2F has been incorrectly titled. The correct title is “Failure-free survival high burden M1”.

