# Supplementary material for: Addition of docetaxel to hormonal therapy in low- and high-burden metastatic hormone sensitive prostate cancer: long-term survival results from the STAMPEDE trial
Source: Ann Oncol. 2019 Sep 27;30(12):1992–2003. doi: 10.1093/annonc/mdz396 (PMC6938598; doi:10.1093/annonc/mdz396)
Supplement: mdz396_Supplementary_Data [file mdz396_supplementary_data.zip › TableS2.docx]

Table S2

| **Worst AE grade** |  | **Up to one year*** | | | |  | **After one year*** | | | |
| --- | --- | --- | --- | --- | --- | --- | --- | --- | --- | --- |
|  |  | **Control** | | **Docetaxel** | |  | **Control** | | **Docetaxel** | |
| **Hypersensitivity** |  |  |  |  |  |  |  |  |  |  |
| **0** |  | 708 | 96% | 300 | 91% |  | 443 | 98% | 248 | 98% |
| **1** |  | 22 | 3% | 19 | 6% |  | 8 | 2% | 5 | 2% |
| **2** |  | 2 | <1% | 8 | 2% |  | 2 | <1% | 1 | <1% |
| **3** |  | 1 | <1% | 2 | 1% |  | 0 | 0% | 0 | 0% |
| **4** |  | 1 | <1% | 2 | 1% |  | 0 | 0% | 0 | 0% |
| **5** |  | 0 | 0% | 0 | 0% |  | 0 | 0% | 0 | 0% |
| *Missing* |  | *1* | *n/a* | *1* | *n/a* |  | *1* | *n/a* | *1* | *n/a* |
| **Blood/bone marrow** |  |  |  |  |  |  |  |  |  |  |
| **0** |  | 574 | 78% | 176 | 53% |  | 314 | 70% | 181 | 72% |
| **1** |  | 133 | 18% | 79 | 24% |  | 115 | 26% | 64 | 25% |
| **2** |  | 18 | 2% | 12 | 4% |  | 13 | 3% | 5 | 2% |
| **3** |  | 7 | 1% | 29 | 9% |  | 6 | 1% | 1 | <1% |
| **4** |  | 1 | <1% | 35 | 11% |  | 3 | 1% | 1 | <1% |
| **5** |  | 0 | 0% | 0 | 0% |  | 0 | 0% | 0 | 0% |
| *Missing* |  | *2* | *n/a* | *1* | *n/a* |  | *3* | *n/a* | *13* | *n/a* |
| **Cardiovascular disorder** |  |  |  |  |  |  |  |  |  |  |
| **0** |  | 671 | 91% | 304 | 92% |  | 398 | 88% | 214 | 84% |
| **1** |  | 41 | 6% | 14 | 4% |  | 30 | 7% | 25 | 10% |
| **2** |  | 10 | 1% | 9 | 3% |  | 16 | 4% | 11 | 4% |
| **3** |  | 10 | 1% | 3 | 1% |  | 6 | 1% | 3 | 1% |
| **4** |  | 2 | <1% | 1 | <1% |  | 3 | 1% | 1 | <1% |
| **5** |  | 1 | <1% | 0 | 0% |  | 0 | 0% | 0 | 0% |
| *Missing* |  | *0* | *n/a* | *1* | *n/a* |  | *1* | *n/a* | *1* | *n/a* |
| **Gastrointestinal disorder** |  |  |  |  |  |  |  |  |  |  |
| **0** |  | 480 | 65% | 89 | 27% |  | 271 | 60% | 152 | 60% |
| **1** |  | 186 | 25% | 165 | 50% |  | 132 | 29% | 74 | 29% |
| **2** |  | 53 | 7% | 60 | 18% |  | 33 | 7% | 23 | 9% |
| **3** |  | 12 | 2% | 16 | 5% |  | 13 | 3% | 4 | 2% |
| **4** |  | 2 | <1% | 0 | 0% |  | 4 | 1% | 1 | <1% |
| **5** |  | 0 | 0% | 1 | <1% |  | 0 | 0% | 0 | 0% |
| *Missing* |  | *2* | *n/a* | *1* | *n/a* |  | *1* | *n/a* | *1* | *n/a* |
| **Endocrine disorder** |  |  |  |  |  |  |  |  |  |  |
| **0** |  | 141 | 19% | 94 | 28% |  | 131 | 29% | 72 | 28% |
| **1** |  | 368 | 51% | 144 | 44% |  | 184 | 41% | 86 | 34% |
| **2** |  | 154 | 21% | 69 | 21% |  | 81 | 18% | 71 | 28% |
| **3** |  | 65 | 9% | 23 | 7% |  | 55 | 12% | 25 | 10% |
| **4** |  | 0 | 0% | 1 | <1% |  | 0 | 0% | 0 | 0% |
| **5** |  | 0 | 0% | 0 | 0% |  | 0 | 0% | 0 | 0% |
| *Missing* |  | *7* | *n/a* | *1* | *n/a* |  | *3* | *n/a* | *1* | *n/a* |
| **General disorder** |  |  |  |  |  |  |  |  |  |  |
| **0** |  | 379 | 52% | 66 | 20% |  | 220 | 49% | 119 | 47% |
| **1** |  | 262 | 36% | 153 | 46% |  | 174 | 38% | 102 | 40% |
| **2** |  | 72 | 10% | 93 | 28% |  | 51 | 11% | 25 | 10% |
| **3** |  | 18 | 2% | 17 | 5% |  | 7 | 1% | 7 | 3% |
| **4** |  | 3 | <1% | 2 | 1% |  | 1 | <1% | 1 | <1% |
| **5** |  | 0 | 0% | 0 | 0% |  | 0 | 0% | 0 | 0% |
| *Missing* |  | *1* | *n/a* | *1* | *n/a* |  | *1* | *n/a* | *1* | *n/a* |
| **Hepatic disorder** |  |  |  |  |  |  |  |  |  |  |
| **0** |  | 645 | 88% | 288 | 87% |  | 392 | 87% | 217 | 86% |
| **1** |  | 73 | 10% | 38 | 11% |  | 46 | 10% | 30 | 12% |
| **2** |  | 8 | 1% | 3 | 1% |  | 8 | 2% | 2 | 1% |
| **3** |  | 6 | 1% | 2 | 1% |  | 5 | 1% | 2 | 1% |
| **4** |  | 2 | <1% | 0 | 0% |  | 0 | 0% | 0 | 0% |
| **5** |  | 0 | 0% | 0 | 0% |  | 0 | 0% | 0 | 0% |
| *Missing* |  | *1* | *n/a* | *1* | *n/a* |  | *3* | *n/a* | *4* | *n/a* |
| **Lab abnormalities** |  |  |  |  |  |  |  |  |  |  |
| **0** |  | 579 | 79% | 243 | 73% |  | 344 | 76% | 185 | 74% |
| **1** |  | 114 | 16% | 66 | 20% |  | 95 | 21% | 58 | 23% |
| **2** |  | 19 | 3% | 9 | 3% |  | 7 | 2% | 5 | 2% |
| **3** |  | 17 | 2% | 9 | 3% |  | 3 | 1% | 3 | 1% |
| **4** |  | 5 | 1% | 4 | 1% |  | 2 | <1% | 0 | 0% |
| **5** |  | 0 | 0% | 0 | 0% |  | 0 | 0% | 0 | 0% |
| *Missing* |  | *1* | *n/a* | *1* | *n/a* |  | *3* | *n/a* | *4* | *n/a* |
| **Metabolic & nutritional** |  |  |  |  |  |  |  |  |  |  |
| **0** |  | 653 | 89% | 277 | 84% |  | 397 | 88% | 226 | 89% |
| **1** |  | 69 | 9% | 47 | 14% |  | 49 | 11% | 19 | 7% |
| **2** |  | 9 | 1% | 6 | 2% |  | 7 | 2% | 9 | 4% |
| **3** |  | 2 | <1% | 1 | <1% |  | 0 | 0% | 0 | 0% |
| **4** |  | 0 | 0% | 0 | 0% |  | 0 | 0% | 0 | 0% |
| **5** |  | 0 | 0% | 0 | 0% |  | 0 | 0% | 0 | 0% |
| *Missing* |  | *2* | *n/a* | *1* | *n/a* |  | *1* | *n/a* | *1* | *n/a* |
| **Musculoskeletal** |  |  |  |  |  |  |  |  |  |  |
| **0** |  | 228 | 31% | 97 | 29% |  | 141 | 31% | 82 | 32% |
| **1** |  | 329 | 45% | 153 | 46% |  | 203 | 45% | 92 | 36% |
| **2** |  | 140 | 19% | 64 | 19% |  | 88 | 19% | 64 | 25% |
| **3** |  | 38 | 5% | 14 | 4% |  | 21 | 5% | 16 | 6% |
| **4** |  | 0 | 0% | 3 | 1% |  | 0 | 0% | 0 | 0% |
| **5** |  | 0 | 0% | 0 | 0% |  | 0 | 0% | 0 | 0% |
| *Missing* |  | *0* | *n/a* | *1* | *n/a* |  | *1* | *n/a* | *1* | *n/a* |
| **Nervous system** |  |  |  |  |  |  |  |  |  |  |
| **0** |  | 581 | 79% | 167 | 50% |  | 365 | 81% | 185 | 73% |
| **1** |  | 118 | 16% | 121 | 37% |  | 64 | 14% | 46 | 18% |
| **2** |  | 26 | 4% | 36 | 11% |  | 18 | 4% | 23 | 9% |
| **3** |  | 6 | 1% | 6 | 2% |  | 4 | 1% | 0 | 0% |
| **4** |  | 2 | <1% | 1 | <1% |  | 2 | <1% | 0 | 0% |
| **5** |  | 0 | 0% | 0 | 0% |  | 0 | 0% | 0 | 0% |
| *Missing* |  | *2* | *n/a* | *1* | *n/a* |  | *1* | *n/a* | *1* | *n/a* |
| **Ocular disorder** |  |  |  |  |  |  |  |  |  |  |
| **0** |  | 692 | 94% | 265 | 80% |  | 416 | 92% | 230 | 91% |
| **1** |  | 33 | 5% | 54 | 16% |  | 33 | 7% | 18 | 7% |
| **2** |  | 6 | 1% | 9 | 3% |  | 1 | <1% | 2 | 1% |
| **3** |  | 2 | <1% | 3 | 1% |  | 3 | 1% | 4 | 2% |
| **4** |  | 0 | 0% | 0 | 0% |  | 0 | 0% | 0 | 0% |
| **5** |  | 0 | 0% | 0 | 0% |  | 0 | 0% | 0 | 0% |
| *Missing* |  | *2* | *n/a* | *1* | *n/a* |  | *1* | *n/a* | *1* | *n/a* |
| **Psychiatric disorder** |  |  |  |  |  |  |  |  |  |  |
| **0** |  | 546 | 74% | 224 | 68% |  | 349 | 77% | 183 | 72% |
| **1** |  | 147 | 20% | 87 | 26% |  | 80 | 18% | 56 | 22% |
| **2** |  | 33 | 5% | 18 | 5% |  | 20 | 4% | 15 | 6% |
| **3** |  | 7 | 1% | 2 | 1% |  | 4 | 1% | 0 | 0% |
| **4** |  | 0 | 0% | 0 | 0% |  | 0 | 0% | 0 | 0% |
| **5** |  | 0 | 0% | 0 | 0% |  | 0 | 0% | 0 | 0% |
| *Missing* |  | *2* | *n/a* | *1* | *n/a* |  | *1* | *n/a* | *1* | *n/a* |
| **Renal disorder** |  |  |  |  |  |  |  |  |  |  |
| **0** |  | 422 | 57% | 196 | 59% |  | 256 | 57% | 117 | 46% |
| **1** |  | 208 | 28% | 100 | 30% |  | 123 | 27% | 85 | 33% |
| **2** |  | 89 | 12% | 31 | 9% |  | 64 | 14% | 43 | 17% |
| **3** |  | 15 | 2% | 4 | 1% |  | 9 | 2% | 8 | 3% |
| **4** |  | 1 | <1% | 0 | 0% |  | 1 | <1% | 1 | <1% |
| **5** |  | 0 | 0% | 0 | 0% |  | 0 | 0% | 0 | 0% |
| *Missing* |  | *0* | *n/a* | *1* | *n/a* |  | *1* | *n/a* | *1* | *n/a* |
| **Respiratory disorder** |  |  |  |  |  |  |  |  |  |  |
| **0** |  | 576 | 78% | 192 | 58% |  | 335 | 74% | 178 | 70% |
| **1** |  | 78 | 11% | 60 | 18% |  | 61 | 13% | 46 | 18% |
| **2** |  | 68 | 9% | 63 | 19% |  | 46 | 10% | 24 | 9% |
| **3** |  | 7 | 1% | 14 | 4% |  | 9 | 2% | 4 | 2% |
| **4** |  | 4 | 1% | 2 | 1% |  | 1 | <1% | 1 | <1% |
| **5** |  | 1 | <1% | 0 | 0% |  | 1 | <1% | 0 | 0% |
| *Missing* |  | *1* | *n/a* | *1* | *n/a* |  | *1* | *n/a* | *1* | *n/a* |
| **Skin disorder** |  |  |  |  |  |  |  |  |  |  |
| **0** |  | 593 | 81% | 111 | 34% |  | 374 | 83% | 196 | 77% |
| **1** |  | 119 | 16% | 159 | 48% |  | 64 | 14% | 43 | 17% |
| **2** |  | 20 | 3% | 58 | 18% |  | 12 | 3% | 13 | 5% |
| **3** |  | 1 | <1% | 2 | 1% |  | 3 | 1% | 2 | 1% |
| **4** |  | 0 | 0% | 1 | <1% |  | 0 | 0% | 0 | 0% |
| **5** |  | 0 | 0% | 0 | 0% |  | 0 | 0% | 0 | 0% |
| *Missing* |  | *2* | *n/a* | *1* | *n/a* |  | *1* | *n/a* | *1* | *n/a* |
| **Total**** |  | **735** | **100%** | **332** | **100%** |  | **454** | **100%** | **255** | **100%** |

*Timed from randomisation

** Total numbers shown for safety population, where 29 patients allocated to the Docetaxel Group never started docetaxel treatment and are therefore included in the SOC group for safety reporting. Total numbers also take into consideration numbers of patients described in Table 3 for whom AE data was not collected (i.e. those who did not report AE data after having died or withdrawn from the trial in the first year from randomisation, or those who did not report AEs after disease progression, as specified in the trial protocol.
