# Supplementary material for: Addition of docetaxel to hormonal therapy in low- and high-burden metastatic hormone sensitive prostate cancer: long-term survival results from the STAMPEDE trial
Source: Ann Oncol. 2019 Sep 27;30(12):1992–2003. doi: 10.1093/annonc/mdz396 (PMC6938598; doi:10.1093/annonc/mdz396)
Supplement: mdz396_Supplementary_Data [file mdz396_supplementary_data.zip › TableS1.docx]

Table S1

| **Patient characteristic** |  | **Low burden** | | | |  | **High burden** | | | |
| --- | --- | --- | --- | --- | --- | --- | --- | --- | --- | --- |
|  |  | **Control** | | **Docetaxel** | |  | **Control** | | **Docetaxel** | |
| **Metastatic burden assessed** |  | 238 | 100% | 124 | 100% |  | 320 | 100% | 148 | 100% |
| **Age at rand’n (years)** |  |  |  |  |  |  |  |  |  |  |
| Median |  | 65 | | 66 | |  | 66 | | 65 | |
| IQR |  | 61 – 70 | | 63 – 71 | |  | 59 – 71 | | 60 – 70 | |
| **WHO performance score** |  |  |  |  |  |  |  |  |  |  |
| 0 |  | 191 | 80% | 97 | 78% |  | 214 | 67% | 107 | 72% |
| 1-2 |  | 47 | 20% | 27 | 22% |  | 106 | 33% | 41 | 28% |
| **T stage** |  |  |  |  |  |  |  |  |  |  |
| T0 |  | 1 | <1% | 0 | 0% |  | 0 | 0% | 1 | 1% |
| T1 |  | 4 | 2% | 0 | 0% |  | 5 | 2% | 0 | 0% |
| T2 |  | 22 | 9% | 16 | 13% |  | 34 | 11% | 20 | 14% |
| T3 |  | 147 | 62% | 81 | 65% |  | 168 | 53% | 74 | 50% |
| T4 |  | 49 | 21% | 21 | 17% |  | 80 | 25% | 37 | 25% |
| TX |  | 15 | 6% | 6 | 5% |  | 33 | 10% | 16 | 11% |
| **Nodal status** |  |  |  |  |  |  |  |  |  |  |
| N0 |  | 70 | 29% | 41 | 33% |  | 115 | 36% | 47 | 32% |
| N+ |  | 155 | 65% | 80 | 65% |  | 167 | 52% | 81 | 55% |
| NX |  | 13 | 5% | 3 | 2% |  | 38 | 12% | 20 | 14% |
| **Site of metastases*** |  |  |  |  |  |  |  |  |  |  |
| Bone |  | 169 | 71% | 81 | 65% |  | 316 | 99% | 145 | 98% |
| Liver |  | 0 | 0% | 0 | 0% |  | 12 | 4% | 4 | 3% |
| Lung |  | 0 | 0% | 0 | 0% |  | 22 | 7% | 12 | 8% |
| Nodes |  | 91 | 38% | 45 | 36% |  | 84 | 26% | 33 | 22% |
| Other |  | 23 | 10% | 14 | 11% |  | 15 | 5% | 6 | 4% |
| **Gleason sum score** |  |  |  |  |  |  |  |  |  |  |
| <= 7 |  | 68 | 29% | 32 | 26% |  | 50 | 16% | 19 | 13% |
| 8-10 |  | 158 | 66% | 89 | 72% |  | 223 | 70% | 99 | 67% |
| *Unknown* |  | *12* | *5%* | *3* | *2%* |  | *47* | *15%* | *30* | *20%* |
| **PSA** |  |  |  |  |  |  |  |  |  |  |
| Median |  | 51 | | 48 | |  | 207 | | 209 | |
| IQR |  | 20 – 123 | | 20 – 99 | |  | 56 – 635 | | 69 – 610 | |
| **Time from diagnosis to rand’n (days)** |  |  |  |  |  |  |  |  |  |  |
| Median |  | 78 | | 82 | |  | 64 | | 70 | |
| IQR |  | 61 – 103 | | 61 – 107 | |  | 47 – 84 | | 50 – 89 | |
| **Planned SOC RT**** |  |  |  |  |  |  |  |  |  |  |
| Not planned |  | 215 | 90% | 110 | 89% |  | 308 | 96% | 142 | 96% |
| Planned |  | 23 | 10% | 14 | 11% |  | 12 | 4% | 6 | 4% |
| **Previous treatment** |  |  |  |  |  |  |  |  |  |  |
| No |  | 216 | 91% | 116 | 94% |  | 312 | 98% | 145 | 98% |
| Yes |  | 22 | 9% | 8 | 6% |  | 8 | 2% | 3 | 2% |
| **Pain from PCa** |  |  |  |  |  |  |  |  |  |  |
| Absent |  | 203 | 85% | 101 | 81% |  | 224 | 70% | 99 | 67% |
| Present |  | 28 | 12% | 22 | 18% |  | 92 | 29% | 47 | 32% |
| *Missing* |  | *7* | *3%* | *1* | *1%* |  | *4* | *1%* | *2* | *1%* |
| **Year of randomisation** |  |  |  |  |  |  |  |  |  |  |
| 2008 |  | 8 | 3% | 4 | 3% |  | 11 | 3% | 4 | 3% |
| 2009 |  | 38 | 16% | 21 | 17% |  | 44 | 14% | 18 | 12% |
| 2010 |  | 46 | 19% | 19 | 15% |  | 59 | 18% | 33 | 22% |
| 2011 |  | 61 | 26% | 39 | 31% |  | 96 | 30% | 39 | 26% |
| 2012 |  | 68 | 29% | 33 | 27% |  | 89 | 28% | 45 | 30% |
| 2013 |  | 17 | 7% | 8 | 6% |  | 21 | 7% | 9 | 6% |
| **Total** |  | **238** | **100%** | **124** | **100%** |  | **320** | **100%** | **148** | **100%** |

* Patients may have had more than one site of metastases at baseline, therefore are represented in more than one ‘site of metastases’ category. Percentages shown are per individual site for the total patients in the arm.

** RT was not SOC for M1 patients at the time of the trial, however, SOC RT was reported as planned for a small proportion of patients due to clinical decisions for these individual cases to receive RT to non-prostate locations, or due to mis-reporting of palliative RT.
