# Supplementary figures and images for: Addition of docetaxel to hormonal therapy in low- and high-burden metastatic hormone sensitive prostate cancer: long-term survival results from the STAMPEDE trial
Source: Ann Oncol. 2019 Sep 27;30(12):1992–2003. doi: 10.1093/annonc/mdz396 (PMC6938598; doi:10.1093/annonc/mdz396)

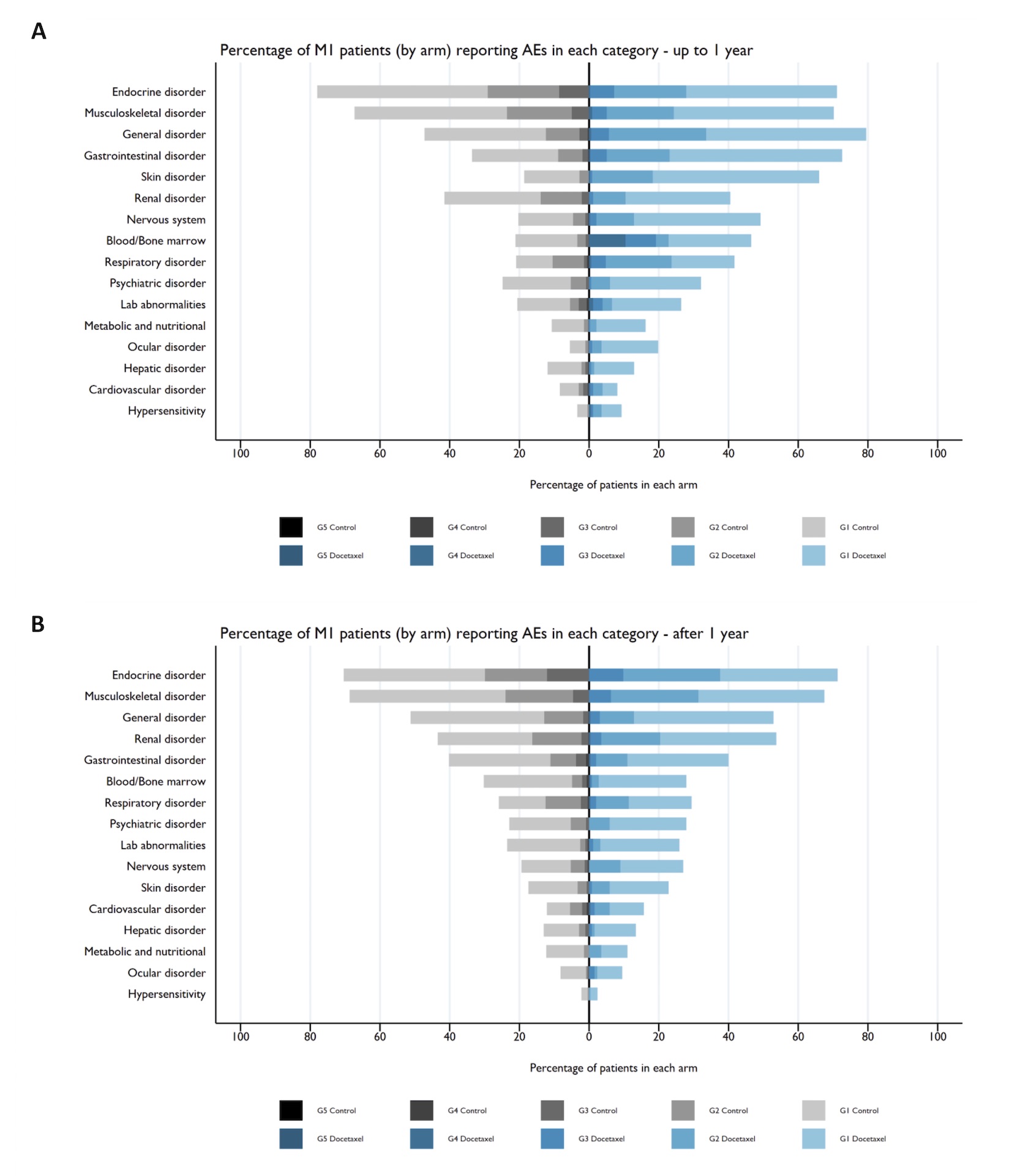

Supplement: mdz396_Supplementary_Data [file mdz396_supplementary_data.zip › FigS1.jpg]
